# Identification of Potential Biomarkers for Liver Cancer Through Gene Mutation and Clinical Characteristics

**DOI:** 10.3389/fonc.2021.733478

**Published:** 2021-09-17

**Authors:** Yunlong Cui, Hua Li, Hongjie Zhan, Tao Han, Yixuan Dong, Caijuan Tian, Yixian Guo, Fang Yan, Dong Dai, Pengfei Liu

**Affiliations:** ^1^Department of Hepatobiliary Oncology, National Clinical Research Center for Cancer, Key Laboratory of Cancer Prevention and Therapy of Tianjin, Tianjin’s Clinical Research Center for Cancer, Tianjin Medical University Cancer Institute and Hospital, Tianjin, China; ^2^Department of Endoscopy, Tianjin Medical University Cancer Institute and Hospital, National Clinical Research Center for Cancer, Key Laboratory of Cancer Prevention and Therapy of Tianjin, Tianjin’s Clinical Research Center for Cancer, Tianjin, China; ^3^Department of Gastric Cancer, National Clinical Research Center for Cancer, Key Laboratory of Cancer Prevention and Therapy of Tianjin, Tianjin’s Clinical Research Center for Cancer, Tianjin Medical University Cancer Institute and Hospital, Tianjin, China; ^4^College of Engineering, Peking University, Beijing, China; ^5^Graduate School, Tianjin Academy of Traditional Chinese Medicine, Tianjin, China; ^6^Tianjin Marvel Medical Laboratory, Tianjin Marvelbio Technology Co., Ltd., Tianjin, China; ^7^Department of Molecular Imaging and Nuclear Medicine, National Clinical Research Center for Cancer, Key Laboratory of Cancer Prevention and Therapy of Tianjin, Tianjin’s Clinical Research Center for Cancer, Tianjin Medical University Cancer Institute and Hospital, Tianjin, China; ^8^Department of Oncology, Tianjin Academy of Traditional Chinese Medicine Affiliated Hospital, Tianjin, China

**Keywords:** liver cancer, circulating tumor DNA (ctDNA), gene mutation, tumor mutation burden (TMB), survival analysis

## Abstract

Liver cancer is a common malignant tumor worldwide, which is a serious threat to the health of people. We try to investigate some mutations and clinical indicators as candidate markers for the development of liver cancer through targeted region capture technology combined with next-generation sequencing. We collected peripheral blood and liver cancer tissue samples from 32 liver patients concurrently. The SeqCap EZ Prime Choice Probe was used to perform the targeted enrichment; this probe captures 1,000 known cancer-associated genes. We calculated the tumor mutation burden (TMB) for each patient. The high-frequency mutations and these relative genes were identified. Eventually, survival analysis was performed based on the mutations and clinical indicators. In 32 liver patients, a total of 29 high-frequency mutations were investigated. They were located in 25 genes, which were enriched in 9 cellular components (CCs), 6 molecular functions (MFs), and 21 biological processes (BPs). Among them, EZH2 c.1544A>G and CCND1 c.839A>T had the highest mutation frequency (5/32). In the protein–protein interaction (PPI) network, EZH2-DNMT3A, NOTCH1-CCND1, and ABL1-CCND1 were the top three pairs. The survival analysis showed that there were significant differences in progression-free survival (PFS) and overall survival (OS) between the Karnofsky performance score (KPS) groups. The PFS and OS in the TMB high group were higher than those in the TMB low group. OS and tumor stage had a remarkable relationship. In conclusion, EZH2 c.1544A>G and CCND1 c.839A>T might be potential biomarkers of liver cancer. TMB might be used as a prognosis and survival indicator of liver cancer.

## Introduction

Cancer is a common disease that poses a threat to human health and affects people’s quality of life. In 2020, liver cancer was one of the cancers with the lowest survival rates (18%), followed by pancreatic cancer (9%). The incidence rate of liver cancer is increasing worldwide; according to the latest cancer statistics, liver cancer has the fastest growth rate among male malignant tumors, with an annual growth rate of 2%–3% (1). Liver cancer can be divided into primary and secondary tumors; primary liver cancer (PLC) can be further divided into hepatocellular carcinoma (HCC), intrahepatic cholangiocarcinoma (ICC), and other rare types (2). The pathogenesis and exact molecular mechanism of liver cancer are not clear.

Mutations in some genes are proven to have a certain relationship with the occurrence and progression of liver cancer (3). For example, Huang and his partners (4) investigated that the base mutation in the p53 gene, which could serve as a novel early diagnostic marker for HCC, provided more information when combined with the usual method of HCC diagnosis. Furthermore, Wong et al. (5) examined that somatic mutations at exon 3 of the β-catenin gene might give rise to inappropriate activation of the Wnt signaling pathway that plays an important role in HCC. Quaas et al. (6) found that analysis of telomerase reverse transcriptase (TERT) promoter mutation might become a diagnostic tool differentiating hepatocellular adenoma (HCA) from well-differentiated HCC and transforming lesions.

Circulating tumor DNA (ctDNA), possessing the initial tumor mutational signature, is released by the tumor cells into the blood, and it reflects the alterations of the gene from primary cancers (7). For cancer patients in whom acquiring biopsy specimens is difficult, ctDNA has become a potential non-invasive cancer biomarker. Nowadays, researchers have found multiple applications for ctDNA in the cancer field. Many studies have demonstrated that ctDNA can be applied for the early detection (8) and monitoring of the minimal residual disease of cancer (9). For instance, Xu et al. (10) constructed a diagnostic prediction model of HCC according to the ctDNA methylation markers, and this model exhibited high diagnostic specificity and sensitivity (p < 0.001). Additionally, ctDNA plays an important role in the evaluation of molecular heterogeneity of overall disease (11) and monitoring of tumor dynamics (12). Ikeda et al. (13) indicated that ctDNA can be used as a marginally invasive alternative method to identify genetic alterations and discover potential molecular therapeutic targets. Furthermore, Cai et al. (14) identified the mutation profiles in biopsy and plasma specimens of HCC patients and found that ctDNA could overcome the heterogeneity of tumor and real-time track the therapeutic responses. Consequently, the clinical application of ctDNA has attracted the attention of more and more researchers. In this study, we systematically analyzed the gene mutations in the liver using targeted region capture combined with next-generation sequencing and identified some potential biomarkers for the occurrence and development of liver cancer.

## Methods

### Patients and Sample Collection

Patients treated at Tianjin Medical University Cancer Institute and Hospital between July 2017 and May 2018 were enrolled. Thirty-two liver patients were collected and named as L01 to L32 successively. Sixteen of the patients were retained from medical records. The ethylene diamine tetraacetic acid (EDTA) tubes were used to collect peripheral blood. Subsequently, the white blood cell and plasma were separated by centrifuging at 2,500*g* for 10 min. To remove any remaining cellular debris, the plasma was further centrifuged at 16,000*g* for 10 min. The plasma supernatant and matched white blood cells were preserved at −80°C separately. In the meantime, the matching tumor tissue samples were gathered. DNA was extracted from tumor tissues and paired white blood cells (control) utilizing the TIANamp Genomic DNA Kit (Tiangen, China). Nevertheless, circulating-free DNA (cfDNA) was extracted from the plasma by the QIAamp Circulating Nucleic Acid Kit (Qiagen, Germany). This study was approved by the Ethics Review Committee of Tianjin Medical University Cancer Institute and Hospital.

### Target Sequencing

The targeted region enrichment of matched tumor samples and blood was accomplished by the SeqCap EZ Prime Choice Probe (Roche, Switzerland), which captures a certain amount of 1.1 Mb from 1,000 known mutation genes of cancer-related genes. Afterwards, the constructed libraries were sent for targeted sequencing using the Illumina HiSeq Xten sequencer at Beijing Novogene Bioinformatics Technology Co., Ltd. (Beijing, China). Average sequencing depths of the tumor samples and blood genomes were approximately 500× and 1,000× respectively.

### Sequencing Data Analysis

The original data were filtered, and the adapter contamination sequences and the low-quality sequences were removed. Subsequently, the clean data were mapped to the hg19 reference genome using Burrows–Wheeler Aligner software (BWA) and analyzed to detect the tumor-specific somatic mutation. To acquire valid mutations, the reads that had exceeded two mismatches were discarded during the alignment process. These mutation sites below 200× in the tumor and below 100× in white blood cells were filtered out. The supporting reads of each mutation site were >3 in the tumor tissue and ≤2 in ctDNA. The concerned statistics of sequencing results are listed in **Supplementary Table S1**.

### Enrichment Analysis

Gene Ontology (GO) terms and Kyoto Encyclopedia of Genes and Genomes (KEGG) pathway enrichment analyses of selected genes were implemented through the Database for Annotation, Visualization, and Integrated Discovery (DAVID) V6.7 (http://david.abcc.ncifcrf.gov/). p < 0.05 was the cutoff standard.

### Construction of the PPI Network

In order to better understand the effect of the selected genes, we constructed the protein–protein interaction (PPI) network by searching the Search Tool for the Retrieval of Interacting Genes (STRING) database (http://string-db.org/). Whereafter, the network was visualized by Cytoscape software.

### Tumor Mutational Burden

Tumor mutation burden (TMB) refers to the number of somatic, coding base substitutions, and short insertions and deletions mutations that occur in the tumor tissue. TMB was calculated by the number of somatic base substitution or insertions and deletions alterations per megabase (Mb) in the coding region target territory of the test. These known somatic and deleterious mutations needed to be removed before calculating TMB. Then, the value was concluded to the exome or genome as a whole. The cutoff values of TMB in this study were obtained from the optimal critical value calculated by Youden’s indexes through receiver operating characteristic (ROC) curves of overall survival (OS) and progression-free survival (PFS).

### Correlation Analysis and Survival Analysis

The clinical data and sequencing results of 16 patients were used for correlation and survival analyses. The Fisher exact test and Mann–Whitney U-test were used to assess the statistical significance. Survival analysis was completed by Kaplan–Meier analysis. When p < 0.05, the results were considered statistically significant.

## Results

### Patients’ Characteristics

Most of the liver cancer patients were men (28/32, 87.50%) with the median age being 53.5 (range, 31–72) years. Among these patients, 15 patients had HCC, 3 patients had ICC, 2 patients had hilar cholangiocarcinoma (HCCA), 3 patients had liver cancer with pulmonary metastasis, and another 9 patients had unknown disease. Because some patients were missing clinical information, only the clinical data of 16 liver cancer patients were analyzed. **Table 1** summarizes the clinical characteristics. Thirteen patients were infected with HBV, and 12 patients had liver cirrhosis. The Karnofsky performance score (KPS) of nine patients was 100, and seven patients was <100 (**Table 1**).

**Table 1:** Clinical characteristics of 16 enrolled liver cancer patients.

Clinical characteristics	No. of patients	Clinical characteristics	No. of patients
Age		HCV infection	
≤52	8	Yes	0
≥52	8	No	14
Gender		Unknown	2
Male	15	Other infections (HPV/HIV, etc.)	
Female	1	Yes	0
Tumor size		No	14
<8 cm	9	Unknown	2
≥8 cm	6	Hypertension	
Unknown	1	Yes	2
KPS		No	12
<100	7	Unknown	2
=100	9	Vascular invasion	
Tumor stage		Yes	6
I stage	1	No	8
I stage	4	Unknown	2
II stage	8	Diabetes	
III stage	3	Yes	4
Liver cirrhosis		No	10
Yes	12	Unknown	2
No	2		
Unknown	2		
HBV infection			
Yes	13		
No	1		
Unknown	2		

KPS, Karnofsky Performance Score; HBV, hepatitis B virus; HCV, hepatitis C virus; HPV, human papillomavirus; HIV, human immunodeficiency virus.

### The Analysis of High-Frequency Mutations in HCC Patients

**Figure 1:**
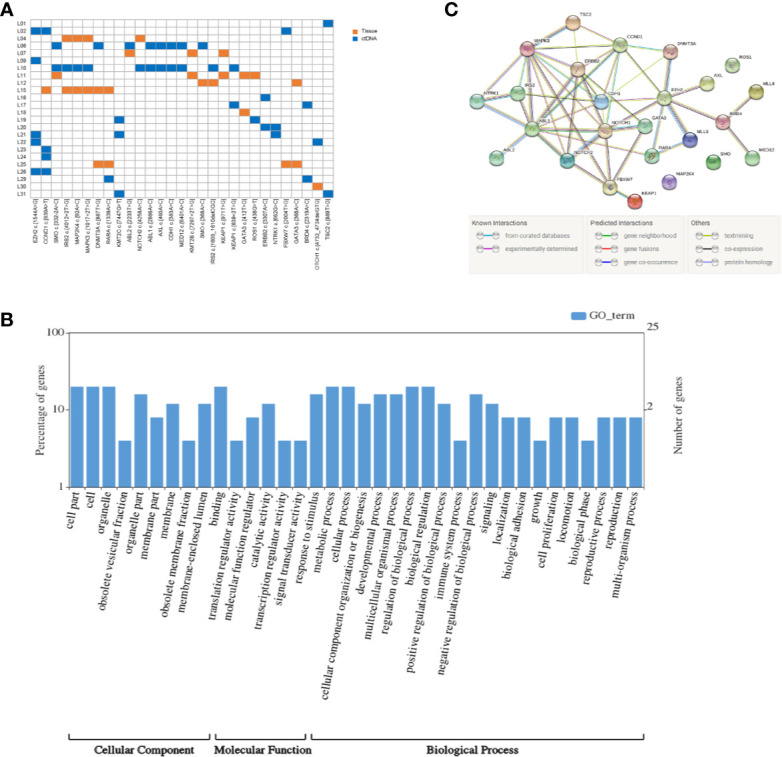
Mutation detection and enrichment of the mutated genes. **(A)** The number of shared mutations found in two or more patients. Each row represents a patient, and each column represents one mutation. Blue indicates that the mutation hotspot was detected in plasma; orange indicates that the mutation was detected in the tissue. **(B)** GO enrichment analysis of genes in which 29 high-frequency mutations are located. **(C)** The PPI networks of these genes in which 29 high-frequency mutations are located.

We integrally analyzed the mutation signature in ctDNA and tumor tissue of every patient. Twenty-nine gene mutations were identified as high-frequency mutations that happened in the lowest two samples (**Figure 1A**). Among them, EZH2 c.1544A>G and CCND1 c.839A>T had the highest frequency (n = 5), and SMO c.332-2A>C, IRS2 c.4012+2T>G, MAP2K4 c.92A>C, MAPK3 c.1017+2T>G, DNMT3A c.887T>G, RARA c.1339A>C, and KMT2C c.7447G>T were detected in three patients; the remaining 20 mutation sites were discovered in two patients. Furthermore, EZH2 c.1544A>G, KMT2C c.7447G>T, ABL1 c.2986A>C, AXL c.460A>C, CDH1 c.383A>C, MED12 c.6401A>C, ERBB2 c.3307A>C, NTRK1 c.662G>C, BRD4 c.2319A>C, and TSC2 c.889T>G only occurred in ctDNA, and KMT2B c.7297+2T>G, KEAP1 c.971T>G, GATA3 c.412T>G, and GATA3 c.368A>C only occurred in tumor tissue. These 29 high-frequency mutation sites were situated in 25 genes. Among these genes, SMO, KEAP1, IRS2, and GATA3 contained two mutation sites, and the residual 21 genes had only one mutation site.

### Enrichment Gene Ontology and Pathway Terms

KEGG pathway and GO enrichment analyses were performed on the above 25 genes. These genes were found to be enriched in 36 GO terms, including 9 cellular components (CCs), 6 molecular functions (MFs), and 21 biological processes (BPs). Among CCs, the mostly enriched terms were cell part, cell, and organelle. In MF, binding was the only term that was mostly enriched. Metabolic process, cellular process, regulation of biological process, and biological regulation were the mostly enriched BPs (**Figure 1B**).

### The PPI Network

Based on the STRING analysis, 50 protein–protein interaction pairs of the above 25 genes were identified (**Figure 1C**). The top 20 pairs that had a high combined score are listed in **Table 2**. Among them, EZH2-DNMT3A, NOTCH1-CCND1, and ABL1-CCND1 were the top three pairs with the highest scores, namely, 0.988, 0.978, and 0.973, respectively.

**Table 2:** The top 20 pairs of the PPI network with high combined score.

Gene1	Gene2	Combined score
EZH2	DNMT3A	0.988
NOTCH1	CCND1	0.978
ABL1	CCND1	0.973
GATA3	NOTCH1	0.969
MAPK3	TSC2	0.965
MAPK3	CDH1	0.961
IRS2	MAPK3	0.950
EZH2	RARA	0.946
NTRK1	IRS2	0.944
NTRK1	ABL1	0.939
ABL2	ABL1	0.936
ERBB2	CCND1	0.931
FBXW7	NOTCH1	0.931
DNMT3A	CCND1	0.924
EZH2	MLL3	0.924
FBXW7	KEAP1	0.911
NOTCH1	NOTCH2	0.908
MLL3	RARA	0.906
NOTCH1	ERBB2	0.901
CDH1	CCND1	0.886

PPI, protein–protein interaction.

### Assessment of Tumor Mutational Burden

**Figure 2:**
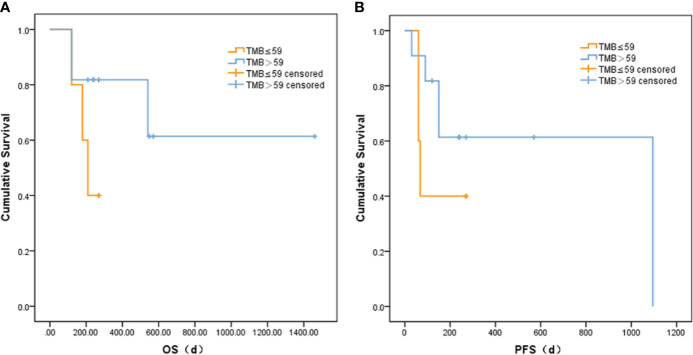
Comparison of **(A)** OS and **(B)** PFS between liver cancer patients with different TMB.

Based on correlation analysis with clinical data, we used the Youden index to select two TMB cutoff values for grouping: 59 mutations/Mb (AUC = 0.583, sensitivity = 0.5, and specificity = 0.8), and 79 mutations/Mb (AUC = 0.547, sensitivity = 0.625, and specificity = 0.625). In the former, there were 9 patients with TMB ≤59 and 23 patients with TMB >59. In the latter, there were 13 patients with TMB ≤79 and 19 patients with TMB >79. When the TMB threshold was 59 and 79, there were significant differences in serum AFP between the low and high group, respectively (p = 0.002 and p = 0.001, **Table 3**). Although there was no statistical difference in the Kaplan–Meier test in the survival analysis, the survival and recurrence times of the TMB >59 mutations/Mb group were significantly longer than those of the TMB ≤59 mutations/Mb group according to the survival curve (**Figure 2**).

**Table 3:** Correlation between TMB and clinicopathological parameters (p-value).

Clinical characteristics	TMB threshold: 59/MB			TMB threshold: 79/MB		
	Low group (0–59/MB)	High group (>59/MB)	p-value	Low group (0–79/MB)	High group (>79/MB)	p-value
Age						
≤52	2	6	1	4	4	1
>53	3	5		4	4	
Liver cirrhosis						
Yes	4	8	0.401	7	5	0.713
No	1	1		1	1	
Tumor size						
<8 cm	3	6	0.604	5	4	0.608
≥8 cm	1	5		2	4	
Tumor stage						
I, II stage	1	4	1	2	3	1
III, IV stage	4	7		6	5	
KPS						
<100	3	4	0.596	4	3	1
=100	2	7		4	5	
AFP	5	11	0.002**	8	8	0.001**

TMB, tumor mutational burden; KPS, Karnofsky Performance Score; AFP, alpha fetoprotein.

**p < 0.001.

### Survival Analysis

**Figure 3:**
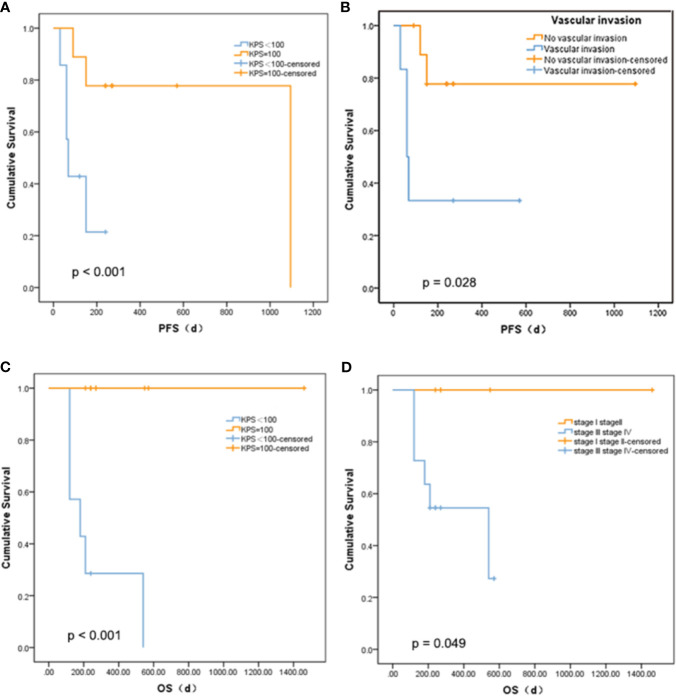
Relationship between survival and clinical indicators. **(A)** Comparison of PFS between liver cancer patients with different KPS. **(B)** Comparison of PFS between liver cancer patients with different vascular invasion status. **(C)** Comparison of OS between liver cancer patients with different KPS. **(D)** Comparison of OS between liver cancer patients with different tumor stage.

There was no statistically significant relationship between PFS and age, diabetes, tumor size, gamma-glutamyl transpeptidase (GGT), alanine aminotransferase (ALT), alkaline phosphatase (ALP), cholinesterase (CHE), or serum albumin (ALB), and OS. The mutations that occur in more than three clinical patients were selected out, namely, CCND1 c.839A>T, EZH2 c.1544A>G, IRS2 c.4012+2T>G, KMT2C c.7447G>T, RARA c.1339A>C, and SMO c.332-2A>C. We did not find a marked relationship between PFS or OS and the presence of these mutations (p = 0.273 and 0.295). Nevertheless, there was a meaningful positive correlation between KPS score and PFS (p < 0.001, **Figure 3A**) or OS (p < 0.001, **Figure 3C**). Patients with a higher KPS scores had longer PFS and OS, and all patients with 100 KPS score survived until the end of the study. Besides, PFS scores were significantly different between patients with and without vascular invasion (p = 0.028, **Figure 3B**). Furthermore, stage I and II liver cancer patients have longer OS than those with stages III and IV (p = 0.049, **Figure 3D**).

## Discussion

In this study, we identified the mutations in liver cancer patients by high-throughput sequencing. EZH2 c.1544A>G and CCND1 c.839A>T were found in five liver cancer patients with the highest mutation frequency. The Enhancer of Zeste Homologue 2 gene (EZH2), a component of polycomb repressive complex 2 (PRC2), is located on human chromosome 7q35. The role of EZH2 gene in tumors, especially in tumor cell invasion and metastasis, has gradually become a research hotspot. EZH2 gene is highly expressed in a variety of malignant tumors but is low or not expressed in normal tissues (15). EZH2 expression is also associated with the malignant features of the tumor and the poor prognosis of the patient (16, 17). Further studies revealed that EZH2 inhibits the expression of ET microRNAs (miRNAs) (EZH2-targeted miRNAs) through the H3K27me3 pathway, thus forming a positive feedback loop of EZH2 miRNAs, maintaining the high expression of EZH2 and promoting the proliferation of tumor cells by regulating key growth inhibitory factors. EZH2 gene participates in the tumorigenesis and progression by promoting cell proliferation, cell cycle arrest, cell migration, and invasion. In addition, EZH2 is the second histone methyltransferase gene that is found to be mutated in cancer. The research found that EZH2 mutations, which cause the change in a single tyrosine in the SET domain of the EZH2 protein (Tyr641), have a high mutation rate in follicular lymphoma (FL), and these mutations were very stable during the development of the disease (18). EZH2 mutation had a certain significance in the development of FL and can represent its early event (19). Studies also found frequent mutations in the EZH2 SET domain [for example, tyrosine residue 646 (Y646)] and EZH2 somatic mutations in various tumors such as germinal center (GC) diffuse large B-cell lymphoma (DLBCL), non-small cell lung cancer, prostate cancer, and colon carcinoma (20). These findings indicate that EZH2 may be a potential therapeutic target in malignant tumors, including liver cancer. Human CCND1 gene encodes the cyclin D1 protein, a regulator of cyclin-dependent kinases (CDKs). At present, CCND1 gene has been recognized as a proto-oncogene whose overexpression can lead to uncontrolled cell proliferation and malignant changes. The CCND1 gene mutation also has been implicated in the development and progression of various cancers. In lung cancer, CCND1 mutation was significantly associated with pathological types and smoking (21). Grünhage et al. (22) indicated that CCND1 c.870A>G mutation was associated with familial colorectal cancer. Although there is no literature report on the impact of EZH2 c.1544A>G and CCND1 c.839A>T on liver cancer, these mutations are pathogenic, and the scores were 0.99 and 0.94 respectively according to the Catalogue of Somatic Mutations in Cancer (COSMIC) database (https://cancer.sanger.ac.uk/cosmic). This indicated that these two mutations have some significance in the study of hepatocellular carcinogenesis.

In order to better understand the interaction between the selected genes, we conducted PPI analysis based on the STRING database. According to the combined score, EZH2-DNMT3A, NOTCH1-CCND1, and ABL1-CCND1 ranked in the top 3 with the scores of 0.988, 0.978, and 0.973, respectively. EZH2 is an element of the epigenetic regulator PRC2 that inhibits gene expression (23). Overexpression of EZH2 is a common case in human cancers and is linked with tumor progression and poor prognosis (12). DNA methylation, one of the most important epigenetic modifications, is essential to gene expression regulation, genomic imprinting, X chromosome inactivation, and tumorigenesis (24). In mammals, DNA methylation patterns are written and regulated by DNA methyltransferases (DNMTs), including DNMT1, DNMT3A, and DNMT3B. Epigenetic damage caused by abnormal DNMTs is related to tumorigenesis, and EZH2 can serve as a recruitment platform for DNA DNMTs. Our result based on the STRING database also showed that there were known interactions and other relationships between EZH2 and DNMT3A. NOTCH proteins (NOTCH1, NOTCH2, NOTCH3, and NOTCH4) play vital roles in embryonic development. Mounting studies indicate that NOTCH is conducive to the pathogenesis of hematopoietic and solid malignancies (25, 26). NOTCH1 mainly engages in angiogenesis and regulates endothelial cell proliferation and migration. The ABL1 proto-oncogene encodes a cytoplasmic and nuclear protein tyrosine kinase that is involved in the processes of cell differentiation, cell adhesion, cytokinesis, and stress response. ABL1 was first discovered as the oncogene in the Abelson murine leukemia virus in the last 38 years (27) and was later recognized as an oncogene participating in chromosomal translocations in human leukemia. The function of CCND1 has been introduced in the previous paragraph. Our result verified that there are clear interactions between NOTCH1 and CCND1, and ABL1 and CCND1 based on data collated from the KEGG databases and others. However, the specific correlations of these genes need to be further studied in subsequent research.

TMB has been regarded as an emerging and isolated biomarker of outcomes with immunotherapy in a variety of tumor types. For instance, Friedlaender et al. (28) showed that plasma TMB could be a dynamic biomarker for immunotherapy treatment in non-small-cell lung cancer (NSCLC). Devarakonda et al. (29) investigated that there was an association between high non-synonymous TMB and the better prognosis in patients with resected NSCLC. Due to the different TMB calculation methods, related thresholds, cancer types, and sequencing panels, there is no uniform division method in the related research fields, and there is less TMB-related literature on liver cancer. The TMB filtration criteria in our study were relatively rough, so the TMB values obtained were high. In the research, the ROC curve based on TMB and OS during follow-up indicated that the best cutoff point was 59 mutations/Mb. Based on the ROC curve of TMB and PFS, the best cutoff point was 79 mutations/Mb. Therefore, we chose 59 and 79 as critical points for grouping and subsequent analysis. Regardless of the critical point of 59 or 79, except for AFP, most of the clinical indicators were not significantly different between the TMB low group and the TMB high group. Moreover, the high-frequency mutations have been shown to be unrelated to patients’ survival. There might be two reasons to explain it. One reason is in the small sample size and the missing clinical information of some patients; only 16 liver cancer patients were further analyzed. The other reason is that the treatments of patients were different. Among the 16 patients, 3 patients underwent radical resection, 3 interventional treatment, 2 radiofrequency therapy, 2 surgery, and the other patients a combination of multiple methods. Our study identified that people with higher TMB had longer overall survival and a better prognosis than those with lower TMB. This meant that the mutated genes and TMB may also act as a biomarker in the development and treatment of liver cancer. However, because the sample size was small in our study, together with the selection of the TMB critical point, further validation of this conclusion is needed in subsequent studies.

The main limitation of this study is that a low number of patients were included; meanwhile, due to the retrospective analysis, clinical information and survival data in some patients are missing, resulting in fewer patients in the survival analysis. Therefore, more cases, biochemical indicators, and additional molecular/cellular experiments are required for a prospective study to verify the results in the future. Hence, although we obtained meaningful results from this study, further research is still necessary.

## Conclusion

Overall, EZH2 c.1544A>G and CCND1 c.839A>T might be novel potential biomarkers of liver cancer. The TMB high group had longer OS or PFS than the TMB low group. Further in-depth studies with large sample sizes are progressing gradually to verify the results and explore their mechanisms on the development of liver cancer.

## Data Availability Statement

The data presented in the study are deposited in the NCBI database repository, accession number PRJNA756802. Further inquiries can be directed to the corresponding authors.

## Ethics Statement

The studies involving human participants were reviewed and approved by Tianjin Medical University Cancer Institute and Hospital. The patients/participants provided their written informed consent to participate in this study.

## Author Contributions

YC, DD, and PL designed the study and helped in preparing the manuscript. HL, HZ, TH, and YD conducted the data mining and collection. CT, YG, and FY conducted the bioinformatics analyses and wrote the manuscript. All authors contributed to the article and approved the submitted version.

## Funding

This study was supported by the Tianjin Natural Science Foundation (grant no. 17JCYBJC25100) and the National Natural Science Foundation of China (grant no. J2024008).

## Conflict of Interest

Author CT, YG and FY were employed by the company Tianjin Marvelbio Technology Co., Ltd.

The remaining authors declare that the research was conducted in the absence of any commercial or financial relationships that could be construed as a potential conflict of interest.

## Publisher’s Note

All claims expressed in this article are solely those of the authors and do not necessarily represent those of their affiliated organizations, or those of the publisher, the editors and the reviewers. Any product that may be evaluated in this article, or claim that may be made by its manufacturer, is not guaranteed or endorsed by the publisher.
