# Supplementary material for: Identification of Potential Biomarkers for Liver Cancer Through Gene Mutation and Clinical Characteristics
Source: Front Oncol. 2021 Sep 17;11:733478. doi: 10.3389/fonc.2021.733478 (PMC8484954; doi:10.3389/fonc.2021.733478)
Supplement: Supplementary file 1 [file DataSheet_1.pdf]

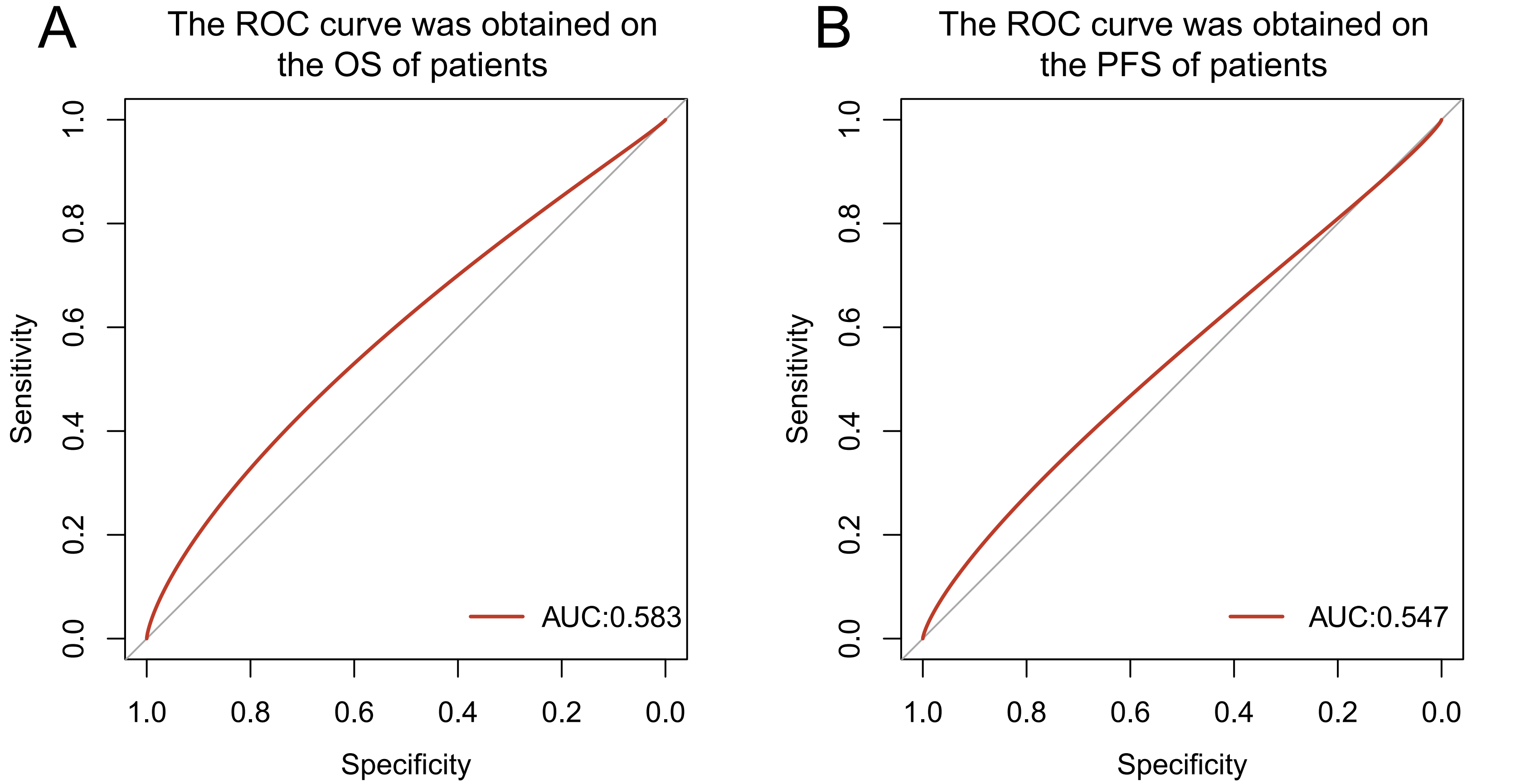
A
The ROC curve was obtained on the OS of patients
Sensitivity
Specificity
1.0
0.8
0.6
0.4
0.2
0.0
AUC:0.583
B
The ROC curve was obtained on the PFS of patients
Sensitivity
Specificity
AUC:0.547
